# Nutritional status of refugee children living in temporary settlements in Europe and MENA region: a systematic review and meta-analysis

**DOI:** 10.1007/s00431-023-04999-x

**Published:** 2023-05-23

**Authors:** Hanaâ Benjeddi, Derre Kwee, Mariken Gruppen, Martijn van der Kuip, Michaël Boele van Hensbroek, Marceline Tutu-van Furth

**Affiliations:** 1 Emma Children’s Hospital, Amsterdam University Medical Centers, Postbus 22660 1100 DD, Amsterdam, The Netherlands; 2 University of Amsterdam, Amsterdam, The Netherlands

**Keywords:** Refugee camps, Refugee, Asylum seekers, Nutritional status, Growth status, Children

## Abstract

An estimated 41% of all forcibly displaced people are children [1]. Many of these children may live in refugee camps, under poor conditions, for years. The health status of children when arriving in these camps is often not recorded, nor is there a good insight into the impact of camp life on their health. We systematically reviewed the evidence concerning the nutritional status of children living in refugee camps in the European and Middle East and North Africa (MENA) regions. We searched Pubmed, Embase, and Global Index Medicus. The primary outcome was the prevalence of stunting, and the secondary outcome was the prevalence of wasting and being overweight. Out of 1385 studies identified, 12 studies were selected, covering 7009 children from fourteen different refugee camps in the Europe and MENA region. There was great heterogeneity among the included studies, which showed that there was a pooled prevalence of stunting of 16% (95% confidence interval 9.9–23%, *I*^2^ 95%, *p* < 0.01) and of wasting of 4.2% (95% CI 1.82–6.49%, *I*^2^ 97%, *p* < 0.01). Anthropometric measurements were done at random points in time during the children’s camp period. However, no study had a longitudinal design, describing the effect of camp life on the nutritional status.

*   Conclusion*: This review showed that there is a relatively high prevalence of stunting and a low prevalence of wasting among refugee children. However, the nutritional status of children when entering the camp and the effect of camp life on their health is not known. This information is critical in order to inform policymakers and to create awareness concerning the health of the most vulnerable group of refugees.

**Table:** 

**What is Known:**
• *Migration is a core determinant of health for children.*
• *There are risk factors at every stage of a refugee child’s journey that lead to compromised health.*
**What is New:**
• *There is a relatively high prevalence of stunting (16%) and a low prevalence of wasting (4.2%) among refugee children living in refugee camps in Europe and the Middle East and North Africa region.*

## Introduction

According to data published in 2022 by the UN Refugee Agency (UNHCR,), for the first time in recorded history, over a hundred million individuals worldwide were forcibly displaced as a result of persecution, conflict, violence, or events seriously disturbing public order [2]. This number has doubled over the last decade. An estimated 41% of all forcibly displaced people are children [1]. Europe and its individual countries have struggled with the high influx of refugees [3].

This inevitably leads to suboptimal solutions where refugees are offered shelter in temporary settlements. These camps were initially designed to keep people safe during specific emergencies, but emergency situations have become protracted, resulting in people living in camps for years [4].

Especially for children, migration is a core determinant of health [5]. The conflict that they flee from affects their health and development in a myriad of ways [6]. The health status of children when arriving in refugee camps is often not recorded, nor is there a good insight into the impact of camp life on their physical and psychological health. This insight is of critical importance, since it is known that the effect of health threats in childhood, such as malnutrition, trauma, and poor living conditions, may have a lasting impact on their health and quality of life. This might be carried into adulthood leading to decreased work productivity [7, 8]. For example, stunting, defined as having a height for age lower than two standard deviations from the WHO Child Growth Standards median, may cause diminished cognitive and physical development, reduced productive capacity, and an increased risk of non-communicable diseases such as diabetes [9, 10]. Suboptimal water, sanitation, and hygiene (WASH) facilities can furthermore lead to poor health [11].

Documenting the health of children on arrival and during their stay in the camps can be challenging because registration systems are not always in place and health care is provided by many different Non-Governmental Organizations (NGOs). Furthermore, children and their families reside in refugee camps for uncertain amounts of time, moving unexpectedly to other camps which makes assessing the impact of camp life on the health status of these children challenging [11].

There are risk factors at every stage of a refugee child’s journey that lead to compromised health. This can be at the pre-migration level (e.g., violence and food insecurity), during the journey (e.g., exposure at sea, hunger, trafficking, acute infectious diseases), and in the country of destination (e.g., suboptimal access to health care, discrimination, caregiver’s mental health problems) [10].

Literature on the health of children in European refugee camps is thought to be limited, but has not been systematically reviewed. The aim of this paper is, therefore, to systematically review the evidence concerning the nutritional status of children arriving and living in refugee camps in Europe and the MENA region. A better understanding of their health status will be of importance to improve policies and awareness concerning the health of the most vulnerable group of refugees.

## Methods

### Search strategy

Our systematic review was registered in PROSPERO under number CRD42022369667. For this systematic review and meta-analysis, a medical information specialist, experienced in systematic reviews, searched the following databases from 1990 to August 2022: Embase, MEDLINE, Global Index Medicus. The Global Index Medicus is a WHO database focused on low- and middle-income countries. We used both controlled terms (i.e., MeSH terms in MEDLINE) and free-text terms related to the nutritional status of refugee children (e.g., growth, nutrition, stunting). For full search strategies, see the appendix. Snowballing and citation coupling was used in order to find additional publications.

### Inclusion and exclusion criteria

We included studies that focused on children within the age range from birth up to 18 years of age and that were performed in refugee camps or comparable settings. We defined these settings as “a temporary settlement meant for displaced people.” In order to have a more in-depth perspective on this topic, the search was extended to the Middle East and North Africa (MENA) region, because many of the refugee children are originally from this region. In addition, camps in the MENA region might be subsequential stops for refugee families. We had no restrictions on the language, date, or publication status. We aimed to include cross-sectional and cohort studies. We excluded studies that entailed health screenings at recipient countries as well as case studies.

### Data extraction

The following information was extracted for each study: author, year of publication, study design, sample size, country or region, and the prevalence of stunting, wasting, and being overweight in percentages including confidence intervals. We chose to include stunting as the main outcome as it is part of the World Health Organization’s Global Nutrition Target for 2025, achieving a 40% reduction in the number of children under 5 who are stunted [12]. Table 1 shows the PICO framework that was used.

**Table 1:** PICO framework describing the population, intervention, control, and outcomes of this systematic review

Population	Refugees or internally displaced children aged 0–18 years
Intervention	Living in a temporary settlement or refugee camps in Europe or MENA region
Control	WHO growth standards [13]
Outcomes	Prevalence of stunting Prevalence of wasting Prevalence of overweight/obese

*MENA* Middle East and North Africa, *WHO* World Health Organization

### Data analysis

Two reviewers (H.B., D.K.) independently screened titles and abstracts of all identified articles for eligibility. Rayyan was used as a screening tool [14]. Additionally, the full-text articles of all potentially relevant studies were retrieved, and eligibility was determined independently by two authors (H.B., D.K.). Differences regarding the inclusion were resolved through discussion. We collected data according to protocol. We contacted study authors twice for clarification of (missing) data in included and potentially eligible studies, specifically requesting data on children in mixed patient cohorts or missing data in abstracts where the article was unavailable. We used the most complete and recent paper if multiple papers assessed the same (sub)population.

### Quality assessment

Quality assessment was performed by utilizing the Joanna Briggs Institute (JBI) critical appraisal checklist for studies reporting on prevalence data. See Appendix 2 (Table 3). We used the Preferred Reporting Items for Systematic Reviews and Meta-Analysis (PRISMA) guidelines. This work was exempted from medical ethical approval as we used data from patients enrolled in studies and trials already approved by relevant ethical committees.

### Outcomes

Our primary outcome was the prevalence of stunting. Stunting was defined as a height for age below 2 standard deviations of the WHO Child Growth Standards median [15]. Secondary outcomes included the prevalence of wasting and obesity. Wasting was defined as a weight-for-height ≤  − 2 SD of the WHO Child growth standards median. Obesity was defined as a weight-for-height ≥  + 2 SD of the WHO Child growth standards median.

### Statistical analysis

Statistical analyses consisted of a pooled analysis using Review Manager 5.4 [16]. Only the studies that reported a confidence interval were used for this analysis. A forest plot was constructed to meta-analyze the primary outcome looking at the prevalence of stunting as well as the prevalence for wasting. If possible, data was stratified for age in 5 years groups based on the published data. The random effects model was used in the meta-analysis. The *I*(2) index was used to assess heterogeneity,

## Results

**Fig. 1:**
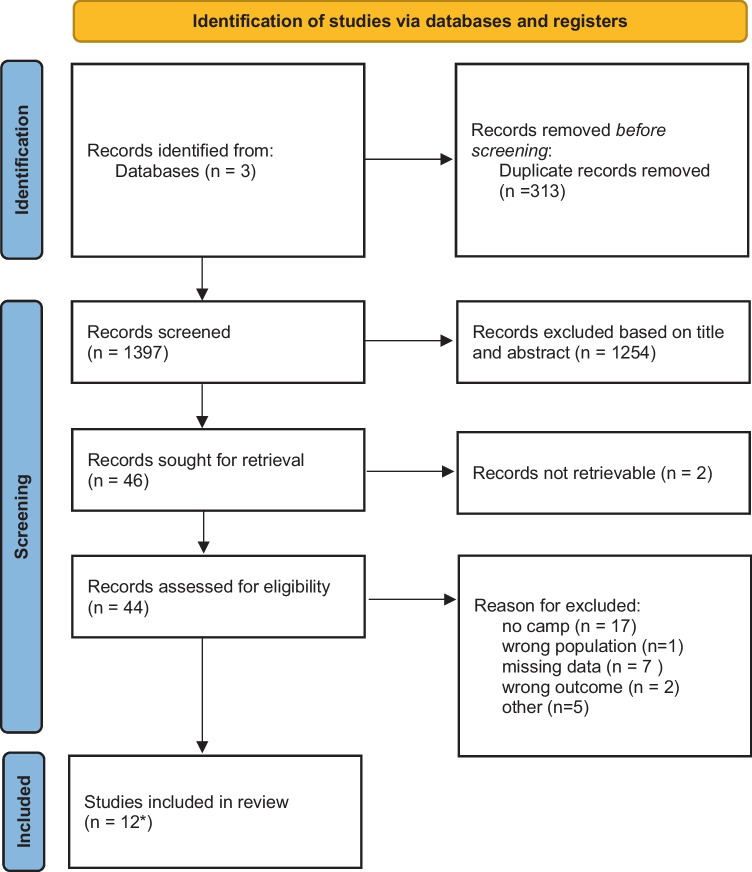
PRISMA flow diagram: nutritional status of refugee children living in camps in Europe and the Middle East and North Africa region. *All included studies are cross-sectional observational studies

This search strategy yielded a total of 1397 studies of which the abstracts were screened, leading to 46 articles that were sought for retrieval. From that list, a definite number of 12 was selected based on the full text. Figure 1 shows the PRISMA flow diagram. The key reason for exclusion was the fact that the study was done in settings other than temporary refugee settings. Table 2 provides details of included studies.

**Table 2:** Characteristics of studies that were included in this systematic review including the location of the refugee camps, the sample size, and prevalences of stunting, wasting, and overweight (prevalence in %, 95% confidence interval)

Studies	Location	Sample size	Stunting (%)	Wasting (%)	Overweight (%)
Grijalva-Eternod et al. [18]	Four Western Sahara refugee camps in Algeria	*N* = 1608	29 (26.4–31.9)	9.1 (7.6–10.7)	2.4 (1.6–3.3)
Hossain et al. [19]	Za’atri refugee camp (Jordan) and Domiz refugee camp (Iraq)	*N* = 1299	16 (11.6–23.4) 19 (15.8–22.7)	1.2 (0.5–3.2) 4.1 (2.8–6.1)	- -
Grammatikopoulou et al. [17]	Two refugee centers in Drama and Kavala, Greece	*N* = 192	7.3 (4.0–11.9)	4.6 (1.0–12.9)	**-**
Bilukha et al. [26]	Za’atri refugee camp (Jordan)	*N* = 327	17 (11.7–24.0)	1.2 (0.5–3.2)	-
Pehlivanturk-Kizilkan et al. [27]	Turkey	*N* = 97	11 (5.9–19.4)	0 (0)	6.0
Walpole et al. [20]	Four refugee camps in Northern Greece	*N* = 177	17 (11.9–24.8)	3.7 (1.4–9.6)	11 (6.3–18.9)
Aakre et al. [28]	Algeria	*N* = 111	13	8.2	-
Khatib et al. [29]	Ruwayshed and Al-Karamah (Jordan)	*N* = 325	12	1.2	-
Kimiagar et al. [30]	9 camps in Iran	*N* = 900	28	16	-
Abukishk et al. [31]	Jerash and Souf refugee camps in Jordan	*N* = 367	23 20	- -	18.2 7.1
El Kishawi et al. [33]	Refugee camp Gaza Strip Palestine	*N* = 217	22	-	-
Haidar et al. [32]	Al-Jad’ah camp in Iraq	*N* = 1389	-	25	-

### Demographics

A total of 7009 children were examined in the 12 included studies. Studies were conducted in Jordan (*n* = 3), Greece (*n* = 2), Algeria (*n* = 2), Turkey (*n* = 1), Iraq (*n* = 1), Iran (*n* = 1), Palestine (*n* = 1), and multiple countries combined (*n* = 1). These children lived in 29 different refugee camp settings. None of the studies reported longitudinal data on the development of the children’s nutritional status during their stay in the refugee camps, i.e., whether their nutritional status improved or worsened over time.

**Fig. 2:**
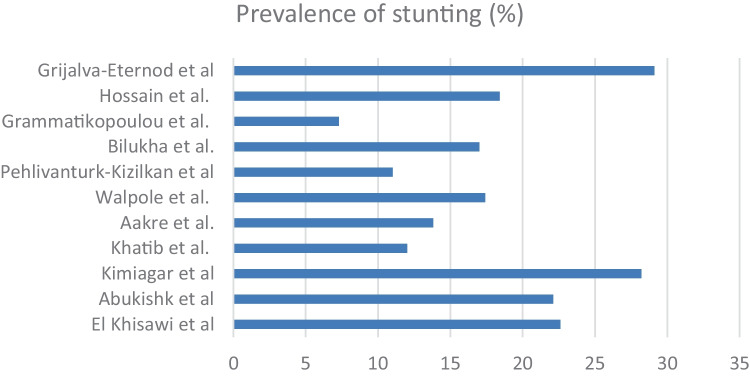
Bar chart showing the prevalence of children being stunted. Prevalence is reported in percentage (%)

The most commonly used methodological approach was using weight, height, and age to construct height-for-age and weight-for-height, from which the prevalence of stunting and wasting was determined. Almost all studies used WHO anthropological definitions to determine the level of wasting and/or stunting. The level of stunting ranged from 7.3 (in Drama and Kavala, Greece [17]) to 29% (in Western Sahara, Algeria [18]). Wasting prevalence ranged from 1.2 (in Za’atari, Jordan [19]) to 5.5% (in four refugee camps in Northern Greece [20]). Figure 2 shows the levels of stunting that were reported.

### Meta-analysis

**Fig. 3:**
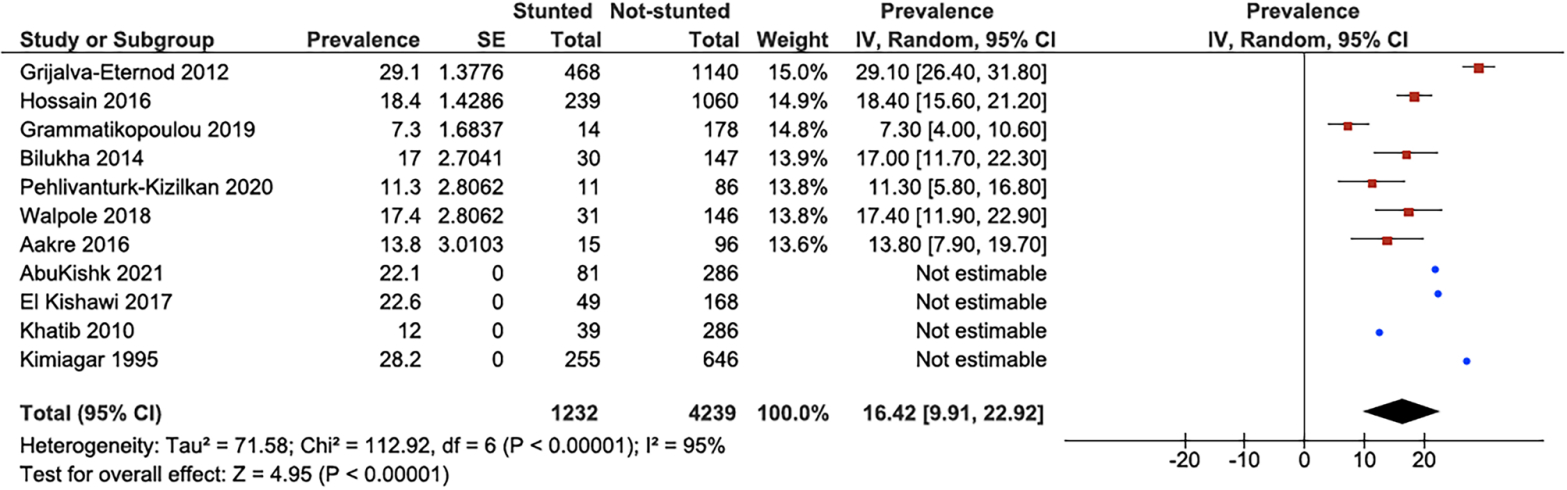
Forest plot showing the prevalence of children being stunted. Only the studies that reported a confidence interval were included in the statistical analysis. The blue dots display studies that reported a prevalence without confidence interval or standard error

All corresponding authors of these studies were contacted in order to complete the data. Three authors provided us with the relevant confidence intervals needed for the meta-analysis. The pooled prevalence of stunting was calculated to be 16.4% with a 95% confidence interval (9.9–23%; *I*^2^ 95%), as can be seen in Fig. 3. There was a high level of heterogeneity, and a random effect was used in the calculation. The heterogeneity was significant between the different studies (*P* < 0.01).

**Fig. 4:**
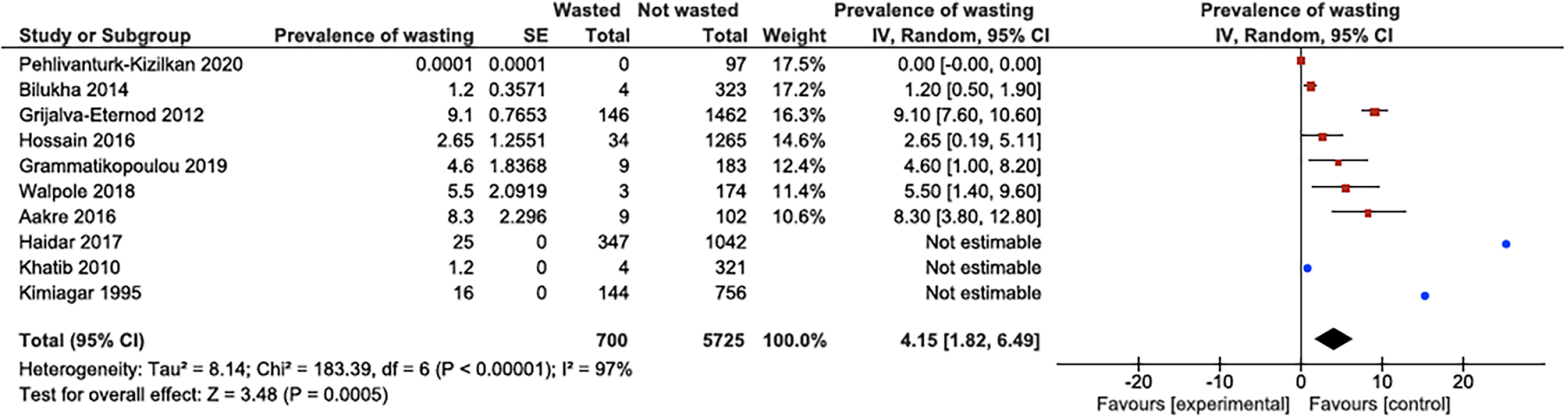
Forest plot showing the prevalence of children being wasted. Only the studies that reported a confidence interval were included in the statistical analysis. The blue dots display studies that reported a prevalence without confidence interval or standard error

The pooled prevalence of wasting was calculated to be 4.2% with a 95% confidence interval (1.82–6.49%; *I*^2^ 97%), as can be seen in Fig. 4. For this calculation, there similarly was a very high level of heterogeneity. The heterogeneity was significantly different between groups (*P* < 0.01).

## Discussion

This systematic review showed that there is a pooled prevalence of stunting of 16% and wasting of 4.2% among refugee children living in temporary settlements in Europe and the MENA region. The number of studies that reported on obesity was too low to allow a meta-analysis on obesity. Interpretation of our findings on the prevalence of wasting and stunting can be done in a multitude of ways.

First of all, ranges of prevalence to classify levels of wasting and stunting, for global monitoring of malnutrition by the World Health Organization, have been used since the 1990s [21]. This classification is used as a tool to prioritize action and guide policymakers. Our review shows that, according to these WHO criteria, out of twelve studies, four were classified as having found a “high,” six a “medium,” and one a “low” prevalence of stunting. The pooled prevalence of stunting in our meta-analysis would be classified as “medium.”

Secondly, compared to the average percentage of stunting in Europe, 4.5% [22], the pooled prevalence of 16% is evidently higher, indicating that children living in refugee camps in Europe and the MENA region are more chronically malnourished when compared to the average child living in Europe.

Conversely, a systematic review that was published in 2021 described the nutritional status of 2,202,869 children (including a small population of refugee children), living in the MENA region [23], and found a pooled prevalence of stunting of 22% (95% CI 20.4–23.6). Compared to this group, our meta-analysis shows that the average prevalence of stunting among children living in European and MENA refugee camps (16%) is lower. It should however be noticed that this MENA region review comprised of a very heterogeneous set of studies in which stunting prevalence varied between 8.7 and 50%. In addition, it is important to realize that the 95% confidence intervals of the two reviews (MENA and ours) overlap. However, an explanation for a difference in prevalence (22% vs 16%) could be that the study population in the 12 studies in our review either have their origins in a country with a lower prevalence of stunting or that their nutritional status improves during their stay in the refugee camps. If the level of stunting found in our review is higher than the level of their country of origin, it might be hypothesized that children arriving in refugee camps have been subject to long and often very stressful journey and chronic food shortage. An example is the prevalence of stunting among Syrian children (8%), as reported in this systematic review, versus the prevalence of 16% as calculated in our meta-analysis. From our review, however, we cannot exclude the possibility that stunting has worsened in the refugee children during their period living in the camps.

The levels of wasting in the twelve studies included in our review can be graded by making use of the WHO framework for malnutrition and results in the following grades: two having a “high,” three a “medium,” one a “low,” and four a “very low” prevalence of wasting. Stunting therefore seems to be more common in children in refugee camps, whereas wasting is very rare. This could be explained by the fact that refugee children are chronically under stress receiving an inadequate diet during their flee to a safer place which often takes months or years, which leads to stunting, rather than acutely and severely deprived of nourishment. Important to note is also the fact that refugee children frequently are born during the mother’s flee, in which period the mother is under stress and might have decreased access to appropriate nutrition. This can lead to intra-uterine malnutrition leading to irreversible stunting in the child’s first years of life [24].

Important to stress again that from the 12 studies included in our review, it is unclear at what point in time the anthropometry was done: on entering the refugee camp or at random points in time during their stay in the camps. It is important when designing effective health interventions to know if these children enter the camp already being stunted or that they become stunted (or wasted) during their stay in the camps. It is known that children in refugee camps can gain weight, reflected as decreasing levels of wasting, during their stay in the camp, as seen in a study done in a large refugee camp in Myanmar where Rohingya refugee children and their families reside [25]. Despite differences in refugee camps, causes of malnutrition and the effect of stressful circumstances could be similar among camps. We therefore also looked at the prevalence of obesity in the included studies; four studies incongruently reported on this, and this parameter therefore was not included in the meta-analysis.

### Limitations

Firstly, the limited number of studies was identified. However, we had a strong search strategy making it unlikely that relevant studies were missed.

Secondly, in designing the review strategy, we became aware that it was challenging to define the concept of a “refugee camp.” Different terms were used in the literature to describe temporary settlements for refugees. The additional complexity is that these temporary settlements turn into long-term solutions, with which the definition of a refugee camp becomes unclear. However, we minimalized this shortcoming by reaching a consensus by discussion in case of doubt (to in- or excluded a study).

Thirdly, next to the challenge of finding a clear definition of a refugee camp, these camps also differ in the way they are set up. There can be differences in terms of WASH facilities, health facilities, and other available services. This limits the generalizability of our study.

## Conclusion

Our review provides evidence for a deprived nutritional status among refugee children living in camps in Europe and the MENA region, as expressed by relatively high levels of stunting. The data in this systematic review does not only contribute to Sustainable Development Goal (SDG) number tree, good health, and well-being: it also contributes to SDG number 16, which aims to promote a peaceful and inclusive society. Our findings highlight the important need for future research to not only assess the health status of children when entering a refugee camp, but also focus specifically on the effect of camp life on the health and nutritional status of refugee children and how it can be improved.

## Data Availability

The data that supports the finding of this study are available from the corresponding author, HB, upon reasonable request.

## Appendix 1

Search terms:

1. Refugees - Refugees [MeSH] OR Refugee Camps [MeSH] OR Refuge* [tiab] OR Refugee Camp* [tiab] OR Refugee cent* [tiab] OR Refugee settlement* [tiab] OR Reception facilit* [tiab] OR Identification center* [tiab] OR displaced person [tiab] OR IDP [tiab] OR asylum seeker [tiab] OR Migrant [tiab] OR Immigrant [tiab]
2. Children - Infant [MeSH] OR Child [MeSH] OR Minor [MeSH] OR Child Development [MeSH] OR unaccompanied minor [tw] OR child*[tw] OR schoolchild*[tw] OR infan*[tw] OR pediatri*[tw] OR paediatr*[tw] OR neonat*[tw] OR toddler*[tw]OR boy[tw] OR boys[tw] OR boyhood[tw] OR girl[tw] OR girls[tw] OR girlhood[tw] OR youth[tw] OR Adolescent [tw] OR Teen [tw]
3. Europe + MENA-region + Turkey - Austria OR Belgium OR Bulgaria OR Croatia OR Cyprus OR Czech Republic OR Denmark OR Estonia OR Finland OR France OR Germany OR Greece OR Hungary OR Ireland OR Italy OR Latvia OR Lithuania OR Luxembourg OR Malta OR Netherlands OR Poland OR Portugal OR Romania OR Slovakia OR Slovenia OR Spain OR Sweden OR Turkey OR Algeria OR Bahrain OR Djibouti OR Egypt OR Iran OR Iraq OR Jordan OR Kuwait OR Lebanon OR Libya OR Morocco OR Oman OR Qatar OR Saudi Arabia OR Palestine OR Syria OR Tunisia OR United Arab Emirates OR Yemen
4. Anthropometrics - Nutritional Status [MeSH] OR Child Nutrition Disorders [MeSH] OR Nutritional inadequacy [tiab] OR Malnutrition [tiab] OR Stunting [tiab] OR Wasting [tiab] OR GAM [tiab] OR SAM [tiab] OR Growth [tiab] OR Underweight [tiab] OR Overweight [tiab] OR Height [tiab] OR Weight [tiab] OR Anthropometric [tiab] OR height-for-age [tiab] OR HAZ [tiab] OR weight-for-height [tiab] OR WAZ [tiab] OR weight-for-age [tiab] OR WFA [tiab] OR Height-for-age [tiab] OR HFA [tiab] OR BMI [tiab] OR BMIZ [tiab] OR MUAC OR mid-upper arm circumference [tiab] OR skinfold thickness [tiab] OR head circumference [tiab] OR waist circumference [tiab].

## Appendix 2

**Table 3:** Joanna Briggs Checklist score

Study	Score
Walpole et al [20]	7
Grammatikopoulou et al [17]	7
Pehlivanturk-Kizilkan et al [27]	6
Hossain et al [19]	8
Bilukha et al [26]	6
AbuKishk et al	9
Khatib et al [29]	8
Grijalva-Eternod et al [18]	9
Haidar et al [32]	4
Kimiagar et al [30	3
El Kishawi et al [33]	8
Aakre et al [28]	8
